# Acute esophageal necrosis: Case report of an unknown entity

**DOI:** 10.1016/j.ijscr.2019.07.041

**Published:** 2019-07-22

**Authors:** A. Maubert, S. Frey, A. Rahili, J. Filippi, E. Benizri

**Affiliations:** a Service de Chirurgie Générale et Cancérologie Digestive, Centre Hospitalier Universitaire de Nice, Hôpital de l’Archet 2, France; b Service de Gastro-Entérologie, Centre Hospitalier Universitaire de Nice, Hôpital de l’Archet 2, France

**Keywords:** Black esophagus, Acute esophageal necrosis, Endoscopy, Esophagus

## Abstract

**Introduction:**

Acute Esophageal Necrosis Syndrome (AENS) is a rare and unknown clinical entity, defined as a diffuse circumferential black-appearing friable esophageal mucosa going from the distal esophageal mucosa to the gastroesophageal (GE) junction. Esophagogastroduodenoscopy (EGD) remains the gold standard in making diagnosis.

**Presentation of case:**

We report here the case of a 45-year-old man with necrosis of the esophagus treated conservatively. Regression of the lesion but persistence of ulcerations were seen on the endoscopic follow-up. Distal esophageal stenosis was then diagnosed and treated by endoscopic dilation.

**Discussion:**

Diagnosis of AENS must be considered when an old patient, with multiple comorbidities, presents an upper digestive hemorrhage. Upper endoscopy is mandatory. Treatment is in most of the cases conservative. Esophageal stenosis is a frequent complication.

**Conclusion:**

Although AENS is a rare clinical entity, it should not be dismissed by doctors, avoiding useless surgical management. This pathology remains nevertheless associated with a considerable mortality rate.

## Highlights

• Acute esophageal necrosis is an unknown entity because it is a rare disease.
• Management including different specialties.
• Time is necessary and surgery is not the only therapy.

## 1 Introduction

AENS, also known as “black esophagus”, is a rare entity diagnosed for the first time by Brennan during an autopsy in 1967 [1]. Goldenberg et al. were the first to characterize it using an endoscope in 1990 [2]. To date, AENS remains an unknown disease world-wide, with no more than a hundred cases reported. Its incidence fluctuates depending on series, going from 0,01% to 0,2% [[3], [4], [5], [6]]. Although this entity is certainly underestimated, its diagnosis is becoming more frequent since the modern era of endoscopy. Circumferentially black esophageal mucosa, stopping abruptly at the GE junction, with or without histologic examination, confirms the diagnosis. The cardia mucosa appears free of necrosis, maintaining its usual color. However, its pathophysiology is unclear and its etiology is most likely multifactorial, combining hemodynamic instability, esophageal backflow of chemical injury and inadequate protective barriers. The prognosis is variable and mostly depends on the patient’s age and his comorbidities. Morbidity and mortality stand high, varying in some series between 32 and 38% [5,7]. We report here a clinical case from our department. This work is respecting the 2018 SCARE criteria [8].

## 2 Case-report

**Fig. 1:**
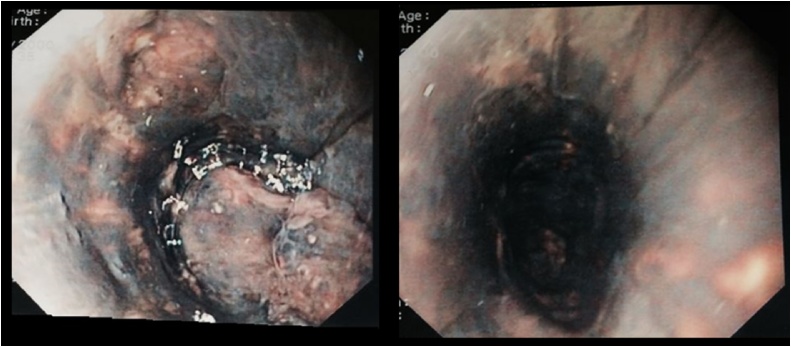
Endoscopic view day 0 with circumferential necrosis of esophagus.

**Fig. 2:**
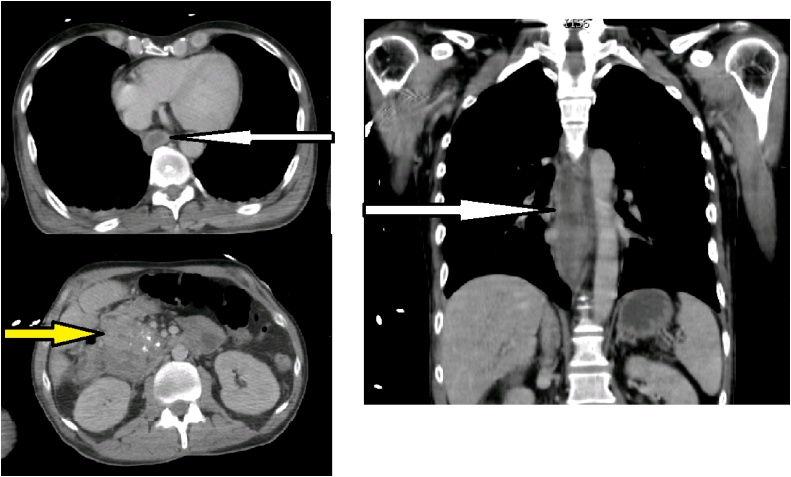
CT-scan with stagnant fluid in esophagus (white arrow) and thickening of the duodenal walls (yellow arrow).

A 45-year-old man, with history of biliopancreatic diversion for alcoholic chronic pancreatitis, was referred to the emergency unit of Pasteur Hospital in Nice for a 72-h history of tiredness, dysphagia and hematemesis. There was no history of peptic ulcer disease or esophageal reflux and he denied ingestion of any caustic agents. On admission, there was no clinical sign of anemia and vital signs were in normal ranges, the patient was afebrile. Blood sample showed an inflammation syndrome with a white blood cell count at 10.8 × 10^9^/L and a CRP measured at 128.0 mg/L. An emergency EGD was then performed and full necrosis of the lower esophagus was diagnosed (Fig. 1). No bleeding site was identified. Microscopically, biopsies reported a full thickness necrosis of the esophagus wall without any extension to sub-mucous or muscular tissues. In the meantime, the patient experienced a cardiac arrest with generalized seizures. He was immediately transferred to the intensive care unit and medical management consisted of sedation, intubation and ventilation. A cervico-thoraco-abdominal computed tomography scan was completed characterizing stagnant fluid all long esophagus, duodenal wall thickening, liquid collection posteriorly to the duodenum and infiltration of fat tissue. No pneumoperitoneum nor pneumomediastinum were seen (Fig. 2). An endoscopic follow-up performed at day one confirmed circumferential necrosis of the esophagus, expanding from 25 of the dental arches to 45 cm, with no complications. The GE junction remained healthy with a sharp transition where the mucosa returns to its normal pink appearance. Biology samples displayed an inflammatory syndrome as well as an acute renal failure with creatinine at 239 μmol/L. Conservative management was decided after a multidisciplinary meeting. Extubation was possible at day 3 and the patient was placed on total parenteral nutrition. The patient was discharged at day 10, with parenteral nutrition and double-dose proton pump inhibitors (PPI). The patient did well and parenteral nutrition was stopped after a total duration of three weeks. Repeated endoscopic follow-up showed a global improvement however residual ulcerations with lower esophageal stricture were seen at one month. Iterative balloon dilation helped improve his clinical condition. Up-to-date, the patient is doing very well and does not require any further endoscopic treatment.

## 3 Discussion

AENS is a rare clinical entity and thus has little mention in literature. This syndrome concerns more often elderly men, with a mean age of 67 years old [6]. The etiology remains uncertain and is mainly multifactorial with the principal factors being low blood flow, diminished mucosal defenses and aggression of the esophageal mucosa by massive gastric content inflow [2,3]. The most common risk factors are heart failure, atherosclerosis, diabetes, renal failure, drugs and cancer. In addition, acute conditions can also be responsible, such as sepsis, congestive heart failure, lactic acidosis, severe bleeding, severe pancreatitis, hypoalbuminemia, hypothermia, low blood pressure or any other trauma or shock [7,9,10]. In our case, the patient was already known for alcoholic chronic pancreatitis, and during his stay, presented a cardiac arrest with severe sepsis and acute renal failure. It is the gathering of these factors that can lead to the development of AENS. The clinical examination often finds hematemesis, thoracic pain and dysphagia, whereas syncope and hyperthermia are not consistent, although it was the case here. Upper endoscopy remains the gold standard in making a diagnosis with a typical appearance of dark esophageal mucous membrane circumferentially (Fig. 1), corresponding to a diffuse necrosis, usually affecting the distal esophagus and migrating proximally progressively covering its total length [[5], [6], [7]]. The esogastric junction is always respected. The reason why distal esophagus is first to suffer tends to the fact that it is the least vascularized esophageal segment, having its arterial supply from terminal branches of the upper esophagus artery and the left gastric artery [11]. Nevertheless, histologic examination is not mandatory for diagnosis, and generally highlights a necrosed mucous membrane with a possible extension in sub-mucous and muscular membranes, associated to intense inflammatory infiltration and vascular thrombosis. The differentials are other etiologies for black esophagus such as melanosis [12], pseudomelanosis [13], malignant melanoma, acanthosis nigricans or ingestion of caustic substance. Treatment is not standardized but generally correspond to acute care management including intravenous hydration, nil-per-os treatment, long-term parenteral nutrition, abolition of gastric acidity (PPI) and mucous protection (sucralfate). Nonetheless, it is important to also treat the coexisting medical diseases. Improvement is notable after one to two weeks with an normal endoscopic aspect of the esophageal mucous membrane, however, in some cases it can takes up to one month. Complications are infrequent but possible. There are three main complications. The first, esophageal perforation, with an incidence lower than 7% [7], and takes its mechanism from the full thickness necrosis of the esophagus wall. The second, a local infection, often turning to mediastinitis. And the third, esophageal stenosis, usually diagnosed later on, and concerns more than 10% of cases [10]. The later was diagnosed for our patient at one month after full recovery, requiring endoscopic management with iterative dilations. Finally, mortality still reaches up to 6% of cases on average [10] but a higher incidence can be found in literature, varying from 32 to 38% [7]. This high rate can be explained by the seriousness of comorbid illnesses rather than the course of the AENS.

In conclusion, AENS is a rare but very serious disease, with specific etiology remaining unknown and considered multifactorial. Diagnosis of AENS must be considered when an old patient, with multiple comorbidities, presents an upper digestive hemorrhage. Early endoscopic diagnosis is essential in order to allow fast and effective therapeutic care. In absence of complications, treatment is conservative involving proton pump inhibitors along management of all comorbidities. Finally, this entity is associated with a considerable rate of mortality.

## Sources of funding

No.

## Ethical approval

Ethical approval has been exempted.

No ethnical approval.

## Consent

Written informed consent was obtained from the patient for publication of this case report and accompanying images. A copy of the written consent is available for review by the Editor-in-Chief of this journal on request.

## Author contribution

Maubert Alexandre: Conceptualization, Redactor.

Frey Sebastien: Redactor, English Translator.

Rahili Amine: Investigator, Supervisor.

Filippi Jérome: Investigator.

Benizri Emmanuel: Supervisor.

## Registration of research studies

No.

## Guarantor

Maubert Alexandre.

## Provenance and peer review

Not commissioned, externally peer-reviewed.

## Declaration of Competing Interest

None.
